# Formation of the germ-disc in spider embryos by a condensation-like mechanism

**DOI:** 10.1186/s12983-016-0166-9

**Published:** 2016-08-11

**Authors:** Matthias Pechmann

**Affiliations:** University of Cologne, Cologne Biocenter, Zülpicher Str. 47B, 50674 Cologne, Germany

**Keywords:** Spider, *Parasteatoda tepidariorum*, Germ-disc formation, Cell shape changes, Maternal to zygotic transition

## Abstract

**Background:**

Determination of the embryonic body axes is a crucial developmental process in all animals. The establishment of the embryonic axes of spiders has been best studied in the common-house-spider *Parasteatoda tepidariorum*. Here, anteroposterior (AP) polarity arises during germ disc formation; the centre of the germ-disc marks the future posterior pole, and the rim of the disc the future anterior pole of the spider embryo. The centre of the germ disc is also needed for the formation of the cumulus, a group of migratory cells needed to establish dorsoventral (DV) polarity. Thus, both body axes depend on proper germ disc formation and patterning. However, these processes have not been fully analysed at the cellular and molecular level.

**Results:**

Here I present new techniques to stain the cell membranes/outlines in live and fixed spider embryos. I show that the germ-disc is formed from a regular and contiguous blastoderm and that co-ordinated cell shape changes, rather than migration of single cells, are required to drive germ-disc formation in *P. tepidariorum* embryos. Furthermore, I show that the rate of cell divisions within the embryonic and extra-embryonic region is not involved in the rapid establishment of the germ-disc. Finally, I show that the process of germ-disc formation is dependent on the initiation of zygotic transcription.

**Conclusions:**

The presented data provide new insights in to the formation of the germ-disc in spider embryos. The establishment of the germ-disc in *Parasteatoda* embryos is a highly dynamic process that involves wide scale cell-shape changes. While most of the blastodermal cells become cuboidal to form the dense germ-disc, the remaining blastodermal cells stay squamous and develop into huge extra-embryonic, yolk rich cells. In addition, this study shows that the onset of zygotic transcription is needed to establish the germ-disc itself, and that the mid-blastula transition of *Parasteatoda tepidariorum* embryos is prior to any overt axis establishment.

**Electronic supplementary material:**

The online version of this article (doi:10.1186/s12983-016-0166-9) contains supplementary material, which is available to authorized users.

## Background

A process that is common to all bilaterally symmetric animals is the establishment of the main body axes (AP and DV). It has been shown that many different mechanisms can drive this key event in different organisms. Determination of the embryonic axes via gravity (present in the chicken) or via the point of sperm entry (present in nematodes or amphibians) are both typical text book examples [1]. Amongst arthropods, axis determination has been studied in detail in the fruit fly *Drosophila melanogaster*. In this system, the maternal localisation of developmental factors is crucial to lay down the orthogonal axes already during oogenesis [2, 3].

Determination and patterning of the main body axes in a non-insect arthropod species, has been well analysed in the common house spider *Parasteatoda tepidariorum.*

Spiders are basally branching arthropods [4, 5], and during the past 15 years the spider, *Parasteatoda tepidariorum* (formerly known as *Achaearanea tepidariorum* [6], has become a popular organism to study the evolution of developmental processes in arthropods [7, 8]. While several aspects of how the dorsoventral body axis is established in this organism have been revealed via time-lapse microscopy and gene knockdown experiments [9–11], only the patterning processes of the already established AP axis have been analysed so far (e.g. [12–16]).

The initial process of AP axis formation in spiders involves the formation of the germ-disc. This process is one of the most important steps during spider embryogenesis as the centre of the germ-disc will become the posterior pole and the rim of the disc will give rise to the anterior part of the spider embryo. The formation of the germ-disc centre, the so-called primary thickening, is of special interest, as the cumulus (a group of migratory cells that are needed to break the radial symmetry of the germ-disc) will develop from this structure [11]. It was shown that in *Parasteatoda japonica* and in *Parasteatoda tepidariorum*, cellularization occurs around the 16 nuclei stage and a regular blastoderm seems to be present shortly before the formation of the germ-disc [17–19]. However, the cellular basis of the process of germ-disc formation and the overall composition of the blastoderm, including the shape of the cells and the connection of the blastodermal cells to each other has been poorly described. It is also unclear whether the process of embryo formation in *P. tepidariorum* is based on single-cell migration, cell shape changes or a combination of both.

**Fig. 1:**
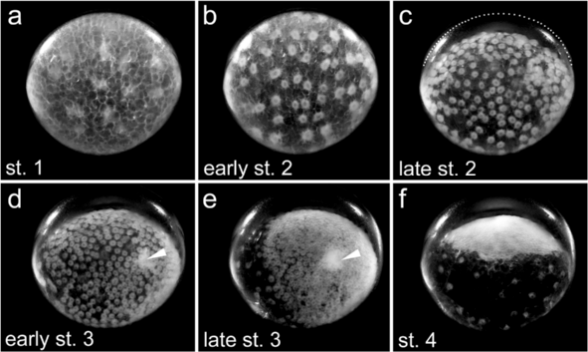
Early developmental stages of a *Parasteatoda tepidariorum* embryo in a side view. After fertilisation, energid cleavages (nuclei with attached perinuclear cytoplasm) occur in the centre of the egg (not shown). Cellularization takes place around the 16 nuclei stage and the nuclei with attached perinuclear cytoplasm reach the periphery of the yolk at the end of stage 1 (**a**) and a blastoderm is formed at stage 2 (**b**). The embryo contracts (**c**) and the perivitelline space is visible at late stage 2 (the upper part of the vitelline membrane is indicated by the dotted line in **c**). At the end of stage 2 and the beginning of stage 3 some cells cluster to form the primary thickening in the centre of the germ-disc (arrowhead in **d** and **e**). A dense germ-disc has formed at stage 4 (**f**). All pictures are stills taken from Additional file 1: Movie 1

Early spider embryos are very suitable for bright field live imaging (see Additional file 1: Movie 1 and Additional file 2: Movie 2 and Fig. 1) because of the very prominent appearance of the nuclei with attached cytoplasm (often described as “cleaving energids” during the early stages of embryonic development; (e.g. [8, 9, 18]). In the early embryos of *Parasteatoda* species, the nuclei with attached cytoplasm (perinuclear cytoplasm) are surrounded by big yolk globules ([17, 18], this study]) and the perinuclear cytoplasm serves as a micro compartment that provides a liquid atmosphere to realise metabolic processes inside of the yolk rich cells.

Prior to the development of early blastomeres microinjections in *P. tepidariorum* spider embryos [7, 18] the description of the development of the early spider embryo was solely based on imaging and analysing the behaviour of the “cleaving energids”. However, injections of fluorescent dyes also mainly lead to the labelling of the perinuclear cytoplasm ([15, 18], this study). A marker to label the cell outlines or cell membranes during the formation of the germ-disc has been missing so far.

Different mechanisms can lead to the formation of the blastoderm in different arthropod species. In insects, like the beetle *Tribolium castaneum,* cellularization is synchronized, and the cellularized blastoderm is uniform [20–22]. This is in contrast to blastoderm formation in the locust *Schistocerca gregaria* or the centipede *Strigamia maritima.* While in the locust single cells start to be cellularized and form a scattered blastoderm before the formation of the embryo [23], the blastoderm of the centipede is formed via the migration of thousands of cells [24]. These examples show how the nature of blastoderm formation can vary greatly in different arthropods.

Here I describe the cellular composition of the blastoderm during the formation of the germ-disc of *P. tepidariorum* embryos. I show that germ-disc formation is based on cell shape changes, and is not due to single cell migration. In addition, I show that the activation of zygotic transcription is needed to establish the germ-disc of *P. tepidariorum* spider embryos.

## Results

### Germ-disc formation in *P. tepidariorum*: cell migration vs. cell shape changes

**Fig. 2:**
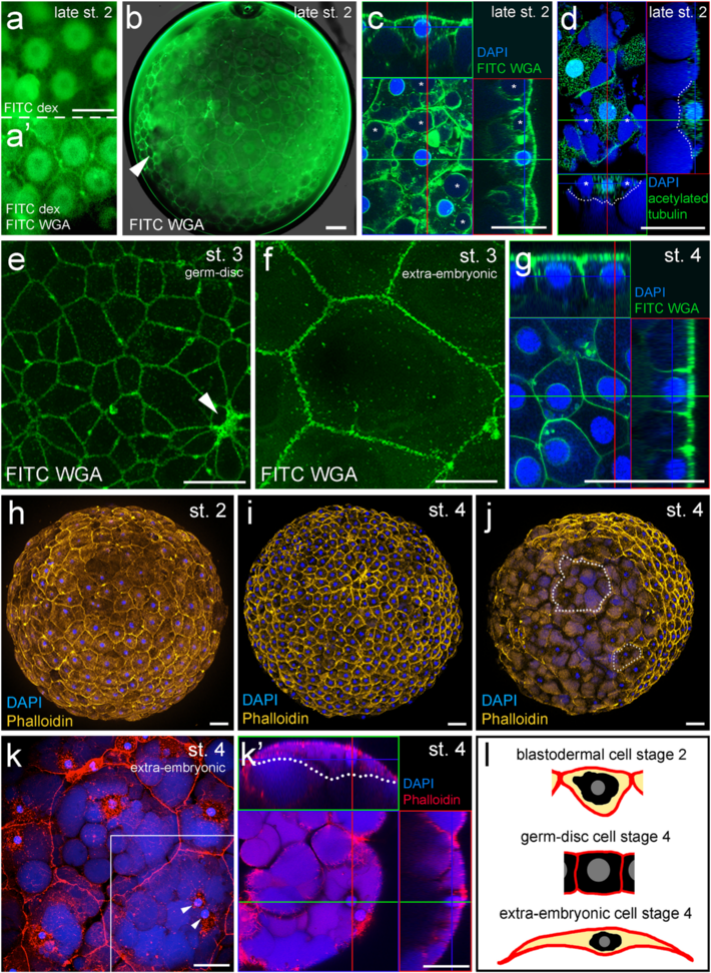
Cell shape analysis of early *P. tepidariorum* embryos via WGA and phalloidin staining. **a** An early stage 1 embryo has been injected with FITC dextran. Several cells have taken up the injected dye and the perinuclear cytoplasm has been labelled. To label the cell membranes, the same embryo has been re-injected with FITC-WGA at late stage 2 (**a’**). **b** Living embryo at late stage 2 stained with FITC-WGA. Arrowhead points towards WGA staining within the vitelline membrane. Single optical slice through a late stage 2 embryo stained for FITC WGA and DAPI (**c**) or acetylated tubulin and DAPI (**d**). As excessive DAPI has not been washed away, the yolk granules are stained, additionally (**d**). Asterisk in **c** and **d** indicate big yolk globules that are inside of the cells. **e** and **f** Confocal scans (maximum intensity projections) of the embryonic (**e**) and extra-embryonic (**f**) cells of living stage 3 embryos that have been injected with FITC WGA. The arrowhead marks the primary thickening in **e**. (**g**) Single optical slice through the germ-disc cells of a stage 4 embryo stained for FITC WGA and DAPI. **h**-**k** Embryos stained with phalloidin to mark actin and DAPI to mark the nucleus of each cell. View on top of the germ-disc is shown in **i**. View on the extra-embryonic region of a stage 4 embryo is shown in **j**. **k** Confocal scan (maximum intensity projection) of a stage 4 embryo that has been stained with phalloidin and DAPI. Two nuclei are present in one cell (arrowheads). (**k’**) Single optical slice through the boxed region shown in **k**. **l** Schematic interpretation of the cells shown in **c**, **g** and **k**'. Cell membranes, red; nuclei, grey; perinuclear cytoplasm, black; yolk, yellow. Dotted lines indicate the cell outlines in **d**, **j** and **k’**. Orthogonal views are boxed in green and red in **c**, **d**, **g** and **k’**. Scale bar is 50 μm in all panels

What is the overall composition of the blastoderm shortly before germ-disc formation? Is a contiguous blastoderm present that requires cell shape changes to form the germ-disc or does germ-disc formation require the migration of single cells? To answer these questions I carried out a search for markers to label the cell outline of *P. tepidariorum* embryos. I found that wheat germ agglutinin (WGA) is a good marker to label the cell membranes of living and fixed spider embryos (Fig. 2, Additional file 3: Movie 3). WGA is known to bind to glycoconjugate proteins that contain N-acetylglucosamine and to sialic acid residues. These residues are predominately found on plasma membranes and WGA has been widely used to stain cell membranes in cell culture and living animals (e.g. [25, 26]).

I injected FITC conjugated WGA to the perivitelline space of *P. tepidariorum* spider embryos. The perivitelline space appears after the contraction of the embryo at the end of stage 2 (Fig. 1c and Additional file 1: Movie 1 and Additional file 2: Movie 2). In living embryos, the injected FITC-WGA often did not distribute evenly within the perivitelline space but stained the cell membranes of the group of cells that were directly underneath the injection point. WGA is endocytosed very quickly and within two hours, a clear cell outline is no longer detectable (Additional file 3: Movie 3). However, as I show here, it is possible to use FITC WGA to follow cell divisions during a short period of time (Additional file 3: Movie 3 and Additional file 11: Fig. S1g-g''). WGA also interacts with the vitelline membrane and stains it in a very regular, soccer ball like pattern (arrowhead in Fig. 2b).

**Fig. 3:**
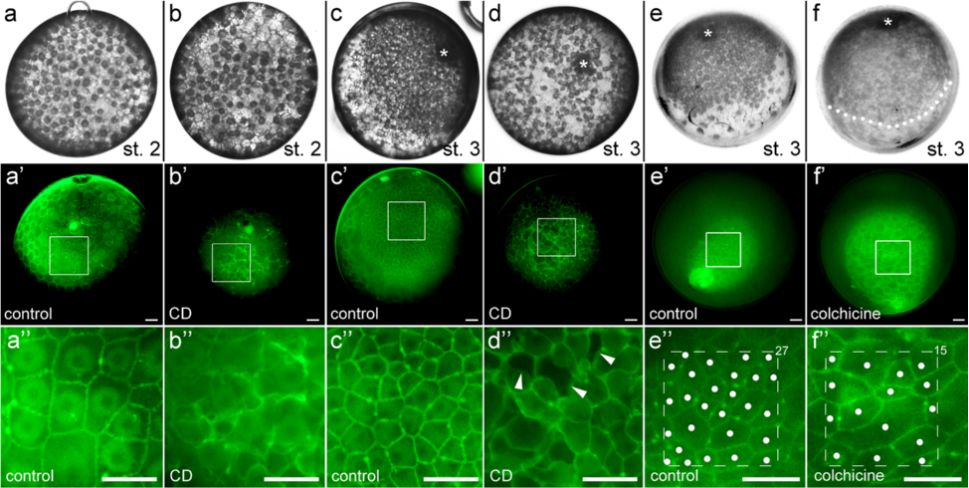
The influence of the actin and microtubule cytoskeleton on the formation of the germ-disc and the integrity of the blastoderm. Stage 2 and 3 control embryos (**a** and **c**) and stage 2 and 3 cytochalasin D injected embryos (**b** and **d**) have been re-injected with FITC-WGA two hours after the first injection. The integrity of the blastoderm (**b'**, **b''**) and of the germ-disc (**d'**, **d''**) is disrupted in CD injected embryos. Arrowheads in **d”** indicate the rupture of the blastoderm. **e**, **f** 5 h after the initial injection and after the formation of the germ-disc (the initial injection was during the course of germ-disc formation at early stage 3), a control embryo (**e**) and a colchicine injected embryo (**f**) have been re-injected with FITC-WGA. Colchicine does not block germ-disc formation (Additional file 6: Movie 6) but blocks further cell divisions (compare the number of cells (indicated by the white dots and the numbers in **e”** and **f”**) within a 100 μm^2^ (indicated by the dashed boxes in **e”** and **f”**)). Boxed areas in **a’** - **f’** are magnified in **a”**- **f”**. The asterisk in **c**-**f** marks the primary thickening. Dotted line in **f** indicates the anterior rim of the germ-disc. The two embryos shown in **a** and **b**, **c** and **d**, **e** and **f** are from the same cocoon, respectively. Scale bar is 50 μm in all panels

FITC-WGA injections into stage 2 embryos revealed that already at this early developmental stage, a contiguous blastoderm is present in *P. tepidariorum* spider embryos (Figs. 2b and 3a' Additional file 11: Fig. S1a and b). Most of the cells have a pentagonal or hexagonal outline (Additional file 11: Fig. S2) and almost all cells are connected to the neighbouring cells at all sites. Only a few (membrane-surrounded) gaps are present between some of the blastodermal cells of the stage 2 embryos (Additional file 11: Fig. S1a).

In general, at this developmental stage the cell membranes are located some distance from the nuclei and its attached perinuclear cytoplasm. This was most evident when I injected FITC-WGA into an embryo in which the cells were already labelled with FITC dextran (via prior injection; compare Fig. 2a, to a’).

FITC-WGA can also be used to stain the cell outline of fixed embryos (Fig. 2c and g). Confocal sectioning of such stained embryos revealed that the blastodermal cells of a stage 2 embryo have a triangular, squamous-like shape (Fig. 2c, l), while the embryonic cells of stage 4 embryos have a cuboidal appearance (Fig. 2g, l).

In addition to the cell membrane, WGA also marked the nuclear envelope (Fig. 2c, g and Additional file 11: Fig. S1f) and other membrane surrounded cell organelles [27–29]. In the germ-disc cells of stage 4 embryos WGA mainly labels the cell outline (Fig. 2g). This is in contrast to the blastodermal cells of late stage 2 embryos in which WGA also surrounds large yolk globules that are present within the cells (Fig. 2c). An antibody staining against acetylated tubulin (Fig. 2d, Additional file 11: Fig. S1d) revealed that microtubules are located near the cell membranes and that the perinuclear cytoplasm is rich in microtubules. In addition, this staining confirms that large yolk globules are present within the cells of late stage 2 embryos (Fig. 2d). Taken together, these results indicate, that the blastodermal cells of a late stage 2 embryo contain yolk, the nuclei and the perinuclear cytoplasm (see schematic in Figs. 2l and 6), and that these blastodermal cells change their shape to form the germ-disc that consists of cuboidal, yolk-less cells.

Another way to mark the outlines of cells is to stain the actin cytoskeleton of fixed spider embryos with phalloidin (Fig. 2h-k). It was already shown that phalloidin can be used to mark the cell outlines of the late germ-disc [9, 14] and I was able to confirm this observation (Fig. 2i). However, it was not shown if the actin cytoskeleton is located near the cell boundaries during earlier stages of development. My staining of late stage 2 embryos shows that already at this early stage, a tight actin network is concentrated near the cell boundaries (Fig. 2h). In addition, phalloidin marks the outline of the large extra-embryonic cells of stage 4 embryos (Fig. 2j, k).

My analysis of both live and fixed embryos, together with findings in the closely related spider *P. japonica* [17], shows that during germ-disc formation the cells of the germ-disc shrink and extrude the yolk (compare Fig. 2c to g, Additional file 4: Movie 4 and Additional file 11: Fig. S3b-b’’’). Already by stage 3 there is a huge size difference between the embryonic and extra-embryonic cells (compare Fig. 2e and f) and by stage 4, a dense germ-disc has formed (Fig. 1f, Additional file 1: Movie 1, Additional file 2: Movie 2, Additional file 3: Movie 3). At this stage, the cells in the centre of the germ-disc have a cone-shaped appearance (Additional file 11: S1e) and form a pore like indentation, which is called the primary thickening [19].

On average, the surface area of a blastodermal cell of a late stage 2 embryo is nearly four times larger than the surface area of a germ-disc cells of a stage 4 embryo (Table 1). In contrast, the surface area of an extra-embryonic cell of a stage 4 embryo is much larger than the surface area of a blastodermal cell of a late stage 2 embryo (on average 4.5 times, Table 1). During germ-disc formation, the cell height also decreases within the embryonic cells (decrease from 30.5 μm, on average, at the highest point of a blastodermal cell of a late stage 2 embryo to 26.1 μm, on average, of a germ-disc cell of a stage 4 embryo, Table 1). In comparison to the blastodermal cells of a late stage 2 embryo, the cell height of the extra-embryonic cells increases only slightly and is very variable (probably dependent on the size of the yolk globules that are present within the cells). In general, the extra-embryonic cells of *Parasteatoda* species are big, yolk rich cells that are very thin at the margins (Fig. 2k’, l, [17]) and the size of these cells is very variable (see Table 1).

**Table 1:** Quantification of the cell surface area and the cell height

	Surface area (μm^2^) *n* = 3;25	Cell height (μm) *n* = 3;10
Stage 2 (embryonic cells)	2365 +/− 777	30.5 +/− 3
Stage 4 (embryonic cells)	604 +/− 144	26.1 +/− 2.7
Stage 4 (extra-embryonic cells)	10649 +/− 6828	32.9 +/− 8.9

Fixed and phalloidin or FITC-WGA stained embryos have been used to measure the cell surface area and height in stage 2 and 4 embryos (n = number of analysed embryos; number of analysed cells)

### The actin cytoskeleton is needed for the integrity of the blastoderm

The combination of WGA, tubulin and phalloidin staining clearly shows that a regular and contiguous blastoderm is present already at stage 2. This finding makes it very unlikely that single cell migration is involved in the process of germ-disc formation. It is more likely that wide scale cell shape changes drive the movement of the future germ-disc cells towards one hemisphere of the embryo. However, to functionally test this hypothesis I injected the actin inhibitor cytochalasin D into embryos that were about to form the germ-disc. After injection of cytochalasin D into early stage 3 embryos, no germ-disc was formed and the cells clustered in big islets (Additional file 5: Movie 5, Additional file 11: Fig S3a-a"). As development continued, large gaps appeared between the islets and yolk granules seem to flow out of these gaps. These observations suggested that the disruption of the actin cytoskeleton led to a disruption of the contiguous blastoderm during the formation of the germ-disc. In addition, I could observe that cytokinesis of cytochalasin D injected embryos did not occur and as a result the cells contained two or more nuclei, several hours after the injection (Additional file 5: Movie 5, compare insets in Additional file 11: Fig. S3a and a’), I used FITC conjugated WGA to clearly show the breakdown of the cellular blastoderm. Indeed, the injection of FITC-WGA into cytochalasin D injected embryos reveals that these embryos have a problem with the integrity of the blastoderm (Fig. 3a-d). In embryos that are about to form the germ-disc (st. 2) and have been injected with cytochalasin D, the cell membranes seem to be arranged very irregularly (compare Fig. 3a” to b”). In stage 4 embryos, holes appear in the already established germ-disc two hours after the injection of cytochalasin D (compare Fig, 3c, c’, c” to d, d’, d”) and cells disperse.

This result indicates that the actin network within the cell is needed for the stability of the contiguous blastoderm and of the already formed germ-disc.

### Analysis of cell division pattern during the formation of the germ-disc

**Fig. 4:**
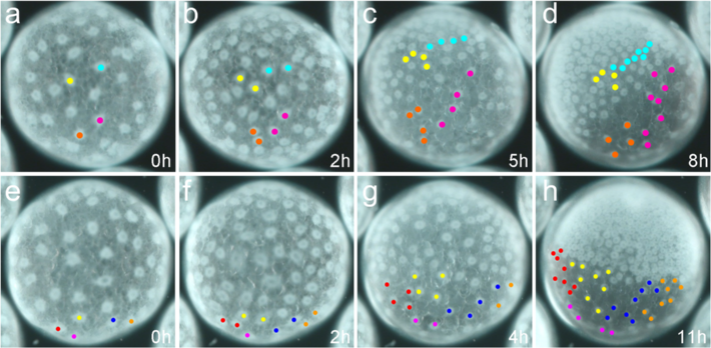
Cell tracking during the formation of the germ-disc. Stills from Additional file 7 and Additional file 8: Movies 7 and 8. **a**-**d** Two cells that will contribute to the germ-disc (yellow and blue) and two cells that will contribute to the extra-embryonic region (orange and magenta) have been analysed for their division rate. During the course of germ-disc formation from late stage 2 to stage 3 the cells divide either two times (resulting in 4 cells; yellow and orange) or three times (resulting in 8 cells; blue and magenta). **e-h** Only the cells that will contribute to the extra-embryonic region of the spider embryo have been tracked from late stage 2 to late stage 3. Please note that the majority of the cells of the early blastoderm (**e**) will contribute to the germ-disc during later stages of development. Time is in hours (h). Only the nuclei with its attached perinuclear cytoplasm have been tracked, as cell outlines are not visible under bright field light conditions

The finding that a contiguous blastoderm is already present at stage 2 leads to the question of whether a higher rate of cell division within the embryonic region could contribute to the rapid establishment of the germ-disc. To analyse the influence of cell divisions on the formation of the germ-disc, I blocked cell divisions via the injection of the microtubule inhibitor colchicine (Fig. 3e and f). In addition, I compared the number of cell divisions of the embryonic and extra-embryonic region during the process of germ-disc formation (Fig. 4a-d).

In contrast to cytochalasin D injections, germ-disc formation is not inhibited after the injection of colchicine into late stage 2 embryos (Additional file 6: Movie 6). However, microtubules seem to stabilize the perinuclear cytoplasm, as it became barely visible after colchicine injection (compare Fig. 3e to f, Additional file 6: Movie 6). Although colchicine injected embryos developed until the germ-disc stage, the injected embryos were not viable and they died shortly after germ-disc formation (see end of Additional file 6: Movie 6).

After the contraction of the embryo (late stage 2, see Fig. 1c) it takes around 6 h to form the germ-disc (see hours 4–10 in Additional file 8: Movie 8). During this period of time most of the cells divide once (see Additional file 7: Movie 7 and Additional file 8: Movie 8). Five hours after colchicine injection, the formed germ-disc possesses around half the amount of cells per 100 μm^2^ (compare Fig. 3e” to f”). Although cell division is blocked in colchicine-injected embryos, the germ-disc forms by incorporating fewer but larger cells. Many of these incorporated cells are elongated along an axis, which is perpendicular to the AP axis of the formed germ-disc (compare Fig. 3e” to f”).

A regular blastoderm without a visible polarity is established around the 64-cell stage (early stage 2, Fig. 1b). Most of the cells of these early stage 2 embryos will contribute to the germ-disc during later stages of development (Fig. 4e-h, Additional file 8: Movie 8). However, comparing the number of cell divisions in the embryonic and extra-embryonic region reveals that there is no difference between these two cell types during the formation of the germ-disc (Fig. 4a-d). In the embryo shown in Fig. 4a-d I tracked two cells of the extra-embryonic region and two cells that will be incorporated to the germ-disc during the course of germ-disc formation (Additional file 7: Movie 7). As already mentioned, I could not observe a difference in cell divisions, and the cells divided either two times (resulting in four cells each, yellow and orange) or three times (resulting in eight cells each, blue and magenta). This analysis shows that germ-disc formation is not triggered by enhanced cell divisions within the cells that will later contribute to the germ-disc. In addition to this finding, tracking the nuclei during the formation of the germ-disc reveals that they can approach each other without any cell divisions (Additional file 9: Movie 9). In some embryos I could observe that extra-embryonic cells tried to divide but then failed, and the nuclei with attached perinuclear cytoplasm fused again (Additional file 11: Fig. S3c-c"). This lead to extra-embryonic cells that contained two nuclei (Fig. 2k, k’ Additional file 11: Fig. S3c-d).

### Analysis of the maternal to zygotic transition (MZT) in *P. tepidariorum*

To analyse whether the activation of the zygotic genome is necessary for germ-disc formation, I injected the RNA Polymerase II inhibitor alpha amanitin (α-AM) to early stage 1 *P. tepidariorum* embryos.

**Fig. 5:**
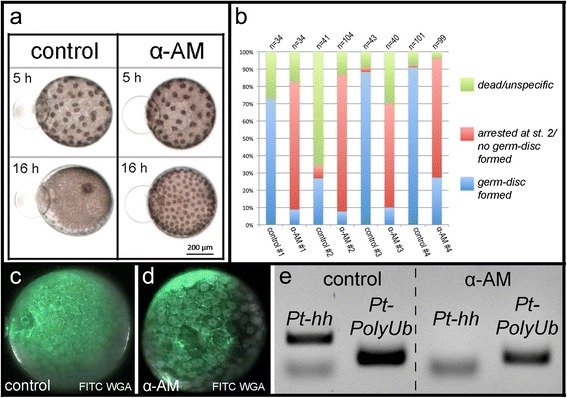
The effect of alpha-amanitin injections on the mid-blastula transition. **a** Stills from Additional file 10: Movie 10. *P. tepidariorum* embryos have been injected with water (control) or alpha amanitin (α-AM) at stage 1. Five hours after injection (5 h) both embryos have proceeded normal embryonic developed. The control embryo has formed a distinct germ-disc 16 h after the injection and has reached stage 4. The alpha-amanitin injected embryo has arrested at late stage 2 and has not formed a germ-disc. **b** Stage 1 embryos of four different cocoons from four different spider females have been injected with water (control #1 - #4) or alpha-amanitin (α-AM #1 - #4). While most of the control embryos develop normally and do form a germ-disc, most of the alpha-amanitin injected embryos do not form a germ disc and have arrested at late stage 2. **c** and **d** The injection of alpha-amanitin does not affect cellularization. **e** PCR for *Pt-hedgehog* (*Pt-hh*) and *Pt-polyubiquitin* (*Pt-PolyUb*) on water (control) and alpha-amanitin (α-AM) injected embryos. While the control gene (*Pt-PolyUb*) is expressed in both samples, the expression of *Pt-hh* is not up regulated in alpha-amanitin injected embryos. See supplementary material for more details

α-AM injected embryos developed normally until stage 2 and contraction of the α-AM injected embryos occurred as in the control injected embryos (Additional file 10: Movie 10 and Fig. 5a). Subsequently, the α-AM injected embryos stopped developing further and were arrested at late stage 2. No germ-disc was established in most of the α-AM injected embryos (Fig. 5b). This result indicates that the activation of zygotic transcription is needed for germ-disc formation and that the mid-blastula transition (MBT, reviewed in [30]) is located at the transition from stage 2 to stage 3. Although further development of the α-AM injected embryos is greatly inhibited, the embryonic cells do not breakdown for several more hours to days (Additional file 11: Fig. S4).

In *Drosophila,* the activation of the zygotic transcription is needed for cellularization [30, 31]. In order to test if in α-AM injected spider embryos the process of cellularization is affected, I injected FITC-WGA to the perivitelline space of control and α-AM injected embryos. α-AM injected embryos show a regular, cellular organisation of the blastoderm (Fig. 5d). In comparison to the control-injected embryos that are advanced in development and have more and smaller cells (Fig. 5c), the cell size of the α-AM injected embryos corresponds to a late stage 2 embryo shortly before germ-disc formation.

In order to confirm that the injection of α-AM blocks the onset of zygotic transcription, I tested whether a gene that is normally up regulated after the MBT is not up regulated after the injection of α-AM. It was previously shown that the gene *Pt-hedgehog* (*Pt-hh*) is not expressed at stage 1 and early 2 but is then up regulated at late stage 2 to stage 3 [32]. By performing a semi-quantitative RT PCR experiment I was able to confirm that *Pt-hh* is not expressed at stage 1 and early stage 2 but is up regulated at stage 3 (Additional file 11: Fig. S5d). Analysing the expression of *Pt-hh* in control and α-AM injected embryos (after RNA extraction from stage 3 control and arrested α-AM injected embryos) revealed that *Pt-hh* is up regulated in the water injected embryos while it is not up regulated in the α-AM injected embryos (Fig. 5e, Additional file 11: Fig. S5e).

## Discussion

Here I show that spider embryos that are about to form the germ-disc show a regular organisation of the blastoderm. By injecting different inhibitors of the cytoskeleton network and by analysing the shape and the composition of the blastodermal, germ-disc and extra-embryonic cells, I show that germ-disc formation is based on cell shape changes, and is not due to single cell migration. In addition, I show that germ-disc formation in *P. tepidariorum* requires the activation of zygotic transcription.

### Germ-disc formation in *Parasteatoda tepidariorum*: a condensation like mechanism

Cellularization and the composition of the forming germ-disc cells has been analysed in a closely related spider species, *Parasteatoda japonica* (formerly known as *Achaearanea japonica* [6]), via electron microscopy [17, 33]. Although these studies show a very detailed analysis of the cellularization process and the fine structure of the single cells, they are missing the overall composition of the blastodermal, extra-embryonic and germ-disc cells. Embryonic development in *Parasteatoda japonica* starts with syncytial cleavages in the centre of the yolk rich eggs. Cell membranes grow from the periphery of the egg and enclose the nuclei and the attached perinuclear cytoplasm at around the 16 nuclei stage. After this cellularization process, the blastomeres surround a blastocoel that has formed in the centre of the egg. The nuclei move to the periphery of the egg and at the same time yolk is sequestered from the blastomeres. This results in large membrane bound yolk granules that can be found below the formed blastoderm. With the contraction of the embryo, blastoderm formation is completed and the blastocoel disappears. The perivitelline space appears and germ-disc formation is initiated.

Here I confirm that a contiguous, cellular blastoderm is present in the embryos of *Parasteatoda tepidariorum* before and during germ-disc formation (Fig. 2). The large blastodermal cells of the stage 2 embryos contain the nuclei, perinuclear cytoplasm and yolk granules. At stage 4, the extra-embryonic cells have increased in size and the surface areas of these cells are, compared to the embryonic cells of the germ-disc, more than 17 times larger (on average, Table 1). At this developmental stage, the embryonic cells have condensed in one hemisphere of the egg and have extruded yolk. While the late stage 2 blastoderm is a squamous epithelium, the embryonic cells of a stage 4 embryo have a cuboidal appearance (Fig. 2c, g).

In *P. japonica* it was shown that most of the blastodermal cells of the germ-disc formation stage (shortly after contraction of the embryo) are around 50 μm in length. It was also reported that the thickness of these cells is around 25 μm in the cytoplasmic region and only 3 μm at the margins [17]. This shows that also in *P. japonica* a squamous epithelium is present shortly before germ-disc formation. My results show that the length and the thickness of the cells of *P. tepidariorum* embryos are comparable to the size of the cells of *P. japonica* embryos at a similar developmental stage (Fig. 2). However, the length of the extra-embryonic cells of a convergence (stage 3) or germ-disc stage (stage 4) embryo in *P. japonica* was stated to be around 100 μm. In principle, this holds also true for *P. tepidariorum* embryos albeit my results show that especially the extra-embryonic cells can vary greatly in size (Fig. 2j, k, and Table 1).

My observations suggest that germ-disc formation does not depend on single cell migration. It seems that there are no migratory cells present in *Parasteatoda* embryos before stage 5. The first migratory cells that can be recognized are the gastrulating cells that enter the germ disc at the primary thickening and at the rim of the germ-disc [7, 9, 15, 34]. Instead, germ-disc formation in *Parasteatoda* embryos seems to occur via a condensation like mechanism. While embryonic germ-disc cells shrink and extrude the yolk, the extra-embryonic cells become large and flat and yolk remains in these cells (this study; [17]). If there is additional uptake of yolk to the extra-embryonic cells remains to be elucidated. The cells of the contiguous blastoderm, which is present at stage 2, have a regular shape and are connected to the neighbouring cells at all sides. Again, this regular shape of the cells suggests that single cell migration is not involved in the process of germ-disc formation. Although the inhibition of the actin cytoskeleton led to cell clustering and blocked germ-disc formation, a closer analysis revealed the breakdown of the contiguous blastoderm (Fig. 3). Actin filaments are enriched at the cell membranes and are probably needed to stabilize the structure of the cells resulting in a stabilisation of the whole blastoderm. A clustering of cells after cytochalasin D treatment was also observed in the crustacean *Parhyale hawaiensis* and it is very likely that actin stabilizes the epithelium of the rosette stage in this organism too [35]. Furthermore, the cellular composition and the localisation of actin at the cell border of the *Parhyale* soccer ball and rosette stage and the *Parasteatoda* blastoderm and germ-disc stage embryos are looking very similar to each other [35]. In addition, germ-disc condensation involving yolk extrusion from the cells that are shortly before gastrulation seems to be a common mechanism in amphipod crustaceans [35–37].

Blastoderm formation in *Parasteatoda* seems to be very different to blastoderm formation in the centipede *Strigamia maritima*. In the latter organism, cellularization occurs in the centre of the big yolk mass and these cells are highly migratory. The cells migrate along the inter-pyramidal spaces of the yolk towards the surface of the egg and several thousands of cells then spread on the surface of the yolk and form the contiguous blastoderm [24]. In *Parasteatoda,* cellularization occurs around the 16 nuclei stage [18]. At this stage the nuclei are located at the deepest side of each blastomere [17] and they reach the surface at stage 2. In spiders plasma threads connect the perinuclear cytoplasm to the periplasm that is located at the surface of the egg and these plasma threads are probably needed to pull the nuclei and its attached cytoplasm towards the surface [17, 38].

Microtubules seem to stabilise the morphology of the perinuclear cytoplasm, as the inhibition of the microtubule network by injecting colchicine results in embryos in which the perinuclear cytoplasm is hardly visible (Additional file 6: Movie 6 and Fig. 3e and f). Also Suzuki and Kondo found microtubules in *P. japonica* that appear along the surface of yolk granules [17] and Schwager and colleagues reported a strong signal for alpha tubulin within the cytoplasmic region of stage 1 and 2 embryos [39].

### Influence of cell divisions on germ-disc formation

There seems to be no difference in the number of cell divisions between extra-embryonic and embryonic cells during the formation of the germ-disc (Fig. 4). Also the injection of cytochalasin D and colchicine does not point towards an influence of cell divisions on the formation of the germ-disc. Although cytochalasin D blocks normal cell divisions as the contractile actin ring that is needed during cytokinesis is missing [40, 41], I found that cytochalasin D injected embryos do not from a germ-disc because of the disruption of the blastoderm. Cell divisions are blocked in colchicine-injected embryos (Fig. 3f). However, germ-disc formation still occurs after colchicine injection and the fewer but larger cells form the germ-disc in one hemisphere of the egg. Again, these results indicate that the formation of the germ-disc is mainly accomplished by a condensation like mechanism in which yolk is extruded from the embryonic cells and that extra-embryonic cells are flattened and stretched during the process of germ-disc formation.

The early subdivision of the embryo into an embryonic and an extra-embryonic (A.K.A abembryonic) region is common to all spiders studied so far. Due to a higher number of cells within the embryonic region, a distinct germ-disc is visible in many higher spider species (e.g. *Parasteatoda tepidariorum*, *Zygiella x-notata*, *Latrodectus spp*.; [9, 42, 43]). In other spider species (e.g. *Cupiennius salei*, [44]) the subdivision into an embryonic and extra-embryonic half becomes first visible after gastrulation when migrating cells do not invade the extra-embryonic hemisphere of the egg and the rate of cell division is higher within the embryonic region. It would be interesting see whether a difference in the rate of cell divisions (between the extra-embryonic and embryonic cells) or if a difference in cell shape changes can explain the differences between spiders that possess and spiders that lack a distinct germ-disc.

### Germ-disc formation is dependent on the onset of zygotic gene expression

The first sign of an asymmetry in *Parasteatoda* embryos is visible shortly after the contraction of the embryo. Some cells cluster and eventually form the primary thickening of the stage 4 embryo. This cell cluster is the centre of the forming germ-disc and most of the cells of the 64-cell stage embryo will contribute to the germ-disc and condense around the future primary thickening (Fig. 4). Here I show that the contraction of the embryo is not affected after injection of the RNA-PolII inhibitor alpha amanitin (Additional file 10: Movie 10). However, germ-disc formation and the formation of the primary thickening are abolished after α-AM injection.

As α-AM has been widely used to block the onset of zygotic transcription (e.g. [31, 45] reviewed in [30]) this result indicates that the MBT is located at the transition from stage 2 to stage 3 and is needed to establish the germ-disc in *P. tepidariorum* embryos. In *Parasteatoda,* cell divisions are synchronous until the embryos have reached the 64-cell stage [18]. The first round of asynchronous cell divisions that leads to the 128-cell stage embryo coincides with the contraction of the embryo. This is a second indicator that the MBT is located at the end of stage 2, as a change in the cell cycle has been reported to be a sign for the MBT in many organisms (reviewed in [30, 46, 47]).

**Fig. 6:**
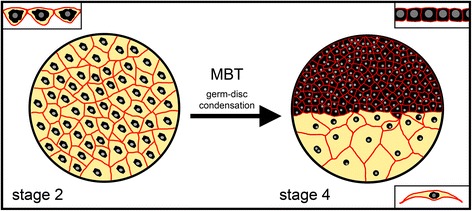
Germ-disc formation in *Parasteatoda tepidariorum*. A contiguous blastoderm is present at stage 2 of embryonic development. The mid-blastula transition (MBT) is at the end of stage 2 and germ-disc formation needs the onset of zygotic gene expression. The germ-disc is formed by a condensation like mechanism. Cell membranes (*red*); nuclei (*grey circles*); perinuclear cytoplasm (*black*); yolk (*yellow*). *Upper left corner*: schematic representation of the cross-section of blastodermal cells (st. 2). *Upper right* corner: schematic representation of the cross-section of germ-disc cells (st. 4). *Lower right* corner: schematic representation of the cross-section of an extra-embryonic cell (st. 4). Not to scale

In *Drosophila* the MBT is needed to complete cellularization [31]. This is not the case in *Parasteatoda* embryos as cell membranes are present in α-AM injected spider embryos (Fig. 5). In *P. tepidariorum* embryos, cellularization occurs already during stage 1 [18] and further normal development probably depends on having a cellularized embryo. Therefore, cellularization in *P. tepidariorum* embryos appears to be a process driven solely by maternally provided material. The results presented here suggest that the germ-disc formation and the establishment of the primary thickening need the onset of zygotic transcription (Fig. 6).

Many different cellular processes are needed to generate the final shape of the germ-disc. First, the cells of the primary thickening need to change their shape to form the pore like indentation. Second, cells need to know whether they are embryonic or extra-embryonic. Third, cell divisions and patterning processes of the germ-disc cells need to continue to build a dense germ-disc that is ready to get transformed into an bilaterally symmetric spider embryo. Therefore, it is very likely that transcription factors, signalling molecules and receptors that receive and transduce patterning information need to be expressed in different parts of the forming germ-disc. My results show that the important patterning gene *Pt-hh* is not up regulated after the injection of α-AM. As a result of the inhibition of the activation of zygotic gene expression the embryo is not able to form a germ-disc.

It has been shown that *Pt-hh* is expressed in the extra-embryonic cells during germ-disc formation and that the receptor *Pt-patched* is expressed within the primary thickening of stage 3 embryos [32]. Hedgehog-signalling has an important role in cumulus migration and in patterning the already established AP axis [32]. Furthermore, it has been suggested that spider Hedgehog could have a role that is analogous to the role of Bicoid in *Drosophila* [18]. However, as already noted by Hilbrant et al., germ-disc formation is not dependent on Hedgehog signalling, as a normal germ-disc with an intact primary thickening is formed after the knockdown of *Pt-hh* and both receptors *Pt-smoothened* and *Pt-patched* [7, 32]. So far, not a single gene has been found that could be involved in the placement of the germ-disc or in the establishment of the primary thickening. Four genes in total have been found that mark the primary thickening and the migrating cumulus cells. These genes are *Pt-decapentaplegic*, *Pt-signed, Pt-hunchback* and *Pt-forkhead* [9, 32, 48]. However, the knockdown of none of these genes resulted in a phenotype in which the primary thickening or the cumulus was not established ([10, 32, 48], unpublished results).

It is interesting to note that the control gene, *Pt-polyubiquitin-C* (*Pt-PolyUb*), is still detectable in α-AM injected embryos. This indicates that there are maternally provided mRNAs present in *P. tepidariorum* embryos, which are not used up during the first two stages of embryonic development. As α-AM is blocking the synthesis of new mRNAs, these maternally provided mRNAs need to have a feature that is leading to an eminent stabilisation of the transcripts. It is known that many maternally provided transcripts are stabilized until the process of maternal clearance leads to the elimination of these transcripts during the MZT (reviewed in [30]).

## Conclusions

The establishment of the germ-disc in *Parasteatoda* embryos is a highly dynamic process that involves wide scale cell-shape changes and requires the onset of zygotic gene expression. Further analysis is needed to reveal which genes are provided maternally or are transcribed zygotically and are needed to the set up the germ-disc and the germ-disc centre in spider embryos.

## Methods

### Spider husbandry and embryology

Embryos and adults of *P. tepidariorum* were obtained from my laboratory stocks in Cologne. Spiders were kept in plastic vials at room temperature and were fed with *Drosophila melanogaster* and crickets (*Acheta domesticus* and *Gryllus bimaculatus*). Embryos were staged according to [19] with minor modifications (subdivision into early and late stages).

### Embryonic Microinjections

Embryos were glued on glass slides using heptane glue and were covered with oil (Voltalef H10S, Atofina). The chorion was not removed. To produce injection needles glass capillaries were pulled using a laser-based micropipette puller P-2000 (Sutter Instrument). The tip of the injection needles were broken with forceps and injections were performed using a micromanipulator and a 50 ml syringe as an injector.

FITC-dextran (Fluorescein isothiocyanate - Dextran 500000-Conjugate; Sigma–Aldrich) injections were performed as described previously ([18], with minor modifications (see above)).

Alpha-amanitin (α–AM, Sigma) was solubilized in water (final concentration 1 μM) and was injected to early (2–5 h after egg lay) stage 1 *P. tepidariorum* embryos. Water injections served as a control.

A 1 % cytochalasin D (Sigma) or 1 % colchicine (Sigma) solution (for both: final concentration 100 μM from a 10 mM ethanol stock) was injected to the perivitelline space of stage 2–4 embryos. As a control a 1 % ethanol solution was used.

Wheat germ agglutinin (WGA, Lectin from *Triticum vulgaris*, FITC conjugate, Sigma) was solved in PBS, diluted 1:5 (from 1 mg/ml stock) and was injected to the perivitelline space of living spider embryos.

### Phalloidin, WGA and antibody staining of fixed embryos

Embryos were fixed as described previously with minor modifications [9]. The vitelline membrane was removed in PBST directly after the fixation. For phalloidin and WGA staining, the embryos were incubated in a DAPI and phalloidin (Molecular Probes, life technologies, 300U, 1 μl/ml) or DAPI and WGA (10 μl WGA/ml PBST from a 1 mg/ml stock) solution. Excessive DAPI, WGA and phalloidin were removed by several washes with PBST.

For antibody staining a monoclonal anti-acetylated tubulin antibody (made in mouse; SIGMA; T7451) was used at a 1:1000 concentration. An Alexa488 goat anti-mouse IgG secondary antibody (ThermoFisher SCIENTIFIC) was used at a 1:400 concentration.

### Semi-quantitative PCR

For the semi-quantitative PCR, the published genome sequence of *P. tepidariorum* [49] has been used to design intron-spanning primers of the genes *Pt-polyubiquitin-C* and *Pt-hedgehog* (accession numbers: XM_016052647.1 (*Pt-PolyUb*); AB125742.1 (*Pt-hh*); see supplemental materials for further details on the experiment and primer sequences).

Total RNA was extracted using TRIzol Reagent (life technologies) and the Quick-RNA MiniPrep Kit (Zymo Research). cDNAs were produced using the RNA to cDNA EcoDry Premix (TaKaRa Clontec, double primed).

### Microscopy, time-lapse imaging and image processing

Pictures were taken using an Axio Zoom.V16 that was equipped with an AxioCam 506 color camera and with a Zeiss AxioImager.Z2 that was equipped with an Apotome.2 module and an AxioCam MRm camera. Confocal imaging was performed on a LSM 700 (Zeiss). Projections of image stacks were carried out using Helicon Focus (HeliconSoft) or Fiji [50]. Live imaging was carried out on the Axio Zoom.V16 and images were processed using Fiji. After confocal sectioning, Fiji has been used to measure the cell surface area and the cell height in fixed embryos that have been stained with WGA or phalloidin.

The embryo shown in Additional file 1: Movie 1 was placed into a cuvette, which was filled with Voltalef H10S oil. The cuvette was placed in front of a 45-degree mirror and embryonic development was recorded by imaging via the mirror.

All movies have been recorded at room temperature and images have been adjusted for brightness and contrast using Adobe Photoshop CS5.

## Abbreviations

α-AM, alpha amanitin; AP, anteroposterior; CD, cytochalasin D; DV, dorsoventral; FITC, fluorescein isothiocyanate; *hh*, *hedgehog*; MBT, mid-blastula transition; MZT, maternal to zygotic transition; *Pt*, *Parasteatoda tepidariorum*; *PolyUb*, *polyubiquitin-C*; WGA, wheat germ agglutinin

## Additional files

Additional file 1: Movie 1. Development of a *Parasteatoda tepidariorum* embryo in a side view. The movie starts at late stage 1 and shows the development until embryonic stage 9. (MOV 8756 kb) Additional file 2: Movie 2. Development of a *Parasteatoda tepidariorum* embryo (starting at stage 1 until the end of stage 8) in a top view. Time is in hours (h). (MOV 1272 kb) Additional file 3: Movie 3. The movie shows the final step of germ-disc condensation. A late stage 3 embryo has been injected with FITC conjugated WGA. The injected WGA marks a region that is located between the primary thickening (marked by the asterisk in B from minute 70 onwards) and the rim of the germ-disc. The same embryo is shown in A (fluorescent signal of the FITC WGA) and B (bright field). Please note that WGA is endocytosed rapidly and marks the cell outline only at the beginning of the movie. (MOV 5670 kb) Additional file 4: Movie 4. A stage 1 embryo has been injected with FITC dextran. This injection did lead to the labelling of a cell clone that was located at the rim of the forming germ-disc. The injected dye marks the perinuclear cytoplasm that surrounds the nuclei and the yolk granules. During germ-disc formation yolk is extruded from the labelled cells. At the end of the movie a dense germ-disc (at stage 4) has formed and no yolk is visible in the FITC dextran labelled cells. The yolk granule is shifted to a more basal position within or is excluded from the germ-disc cell. (MOV 1904 kb) Additional file 5: Movie 5. An early stage 3 embryo has been injected with the actin inhibitor cytochalasin D. Germ-disc formation is inhibited. Cells cluster and normal cell division is inhibited. At the end of the movie the cells contain two or more nuclei. (MOV 7285 kb) Additional file 6: Movie 6. An early stage 3 embryo has been injected with the microtubule inhibitor colchicine. The perinuclear cytoplasm surrounding the nuclei is hardly visible. As indicated by the white line, germ-disc formation is not inhibited. The injected embryo is not viable as the formed germ-disc degenerates over time. (MOV 1865 kb) Additional file 7: Movie 7. Two cells that will give rise to extra-embryonic cells (orange and magenta) and two cells that will contribute to the germ-disc (yellow and blue) have been tracked during the initial phase of germ-disc formation. The tracking starts at the 64 cell stage. Time is in hours (h). (MOV 937 kb) Additional file 8: Movie 8. All cells that will give rise to extra-embryonic cells have been tracked during germ-disc formation. The tracking starts at the 64 cell stage. Only 5 cells will contribute to the extra-embryonic region of the embryo. Please not that only half of the embryonic and extra-embryonic cells are visible during the tracking. Time is in hours (h). (MOV 1470 kb) Additional file 9: Movie 9. Two nuclei have been tracked during the formation of the germ-disc. Although the cells do not undergo cell division during the tracking period the nuclei and surrounding cytoplasm approach each other. Pictures have been taken every minute. (MOV 1012 kb) Additional file 10: Movie 10. While control embryos (injected with water) develop normally, alpha amanitin (α-AM) injected embryos arrest at the end of stage 2 and do not form a germ-disc. Time is in hours (h). (MOV 1401 kb) Additional file 11. Supplementary figures and methods; contains the Figures S1-S5. (PDF 10478 kb)
