# Supplementary material for: Formation of the germ-disc in spider embryos by a condensation-like mechanism
Source: Front Zool. 2016 Aug 11;13:35. doi: 10.1186/s12983-016-0166-9 (PMC4982120; doi:10.1186/s12983-016-0166-9)
Supplement: Additional file 11. — Supplementary figures and methods; contains the Figures S1-S5. (PDF 10478 kb) [file 12983_2016_166_MOESM11_ESM.pdf]

Formation of the germ-disc in spider embryos by a condensation-like mechanism

Matthias Pechmann

University of Cologne, Cologne Biocenter, Zülpicher Str. 47B, 50674 Cologne, Germany

Supplementary Figures and Methods

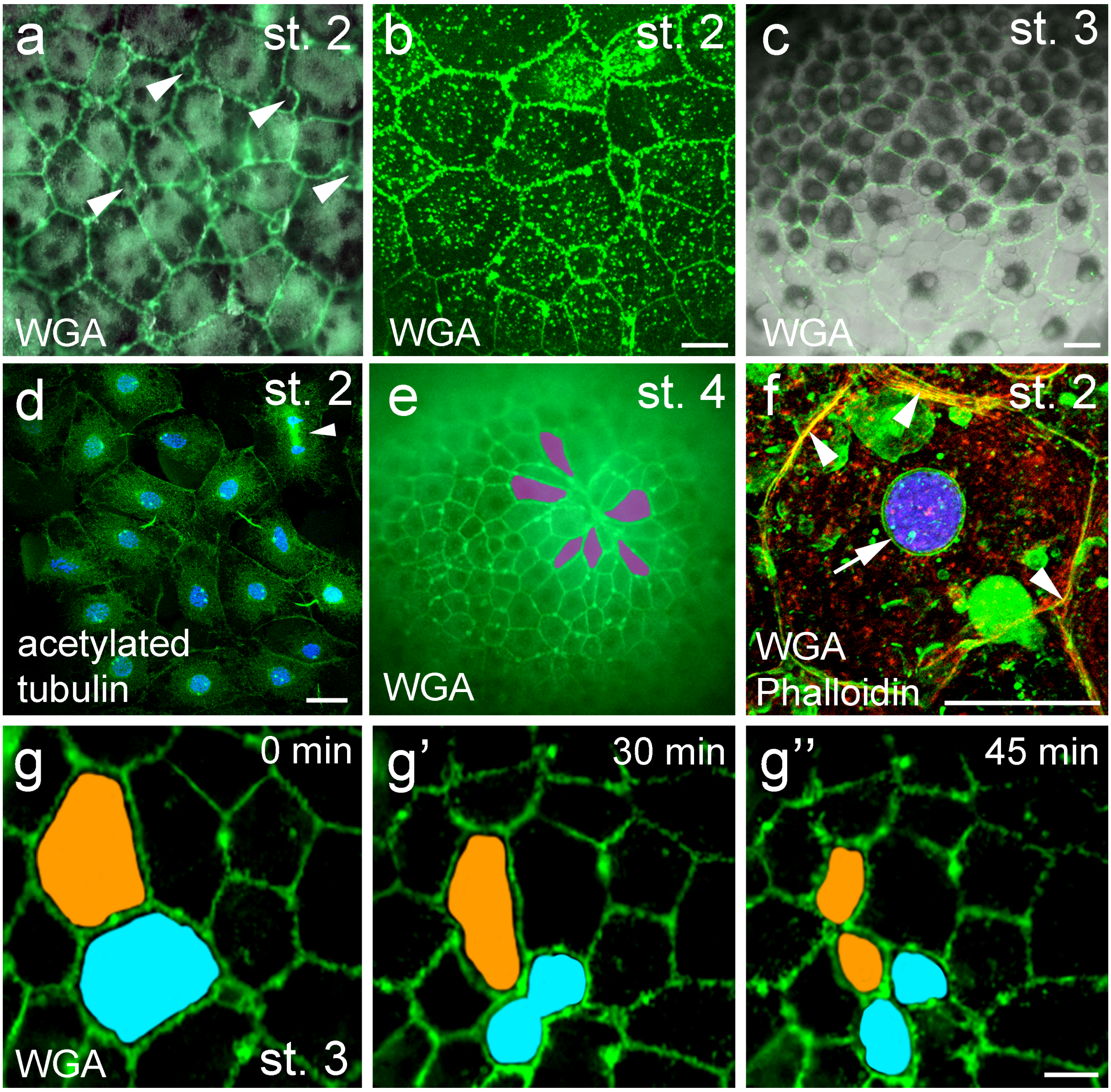

Fig. S1 (**a**) A living stage 2 embryo injected with FITC WGA. Arrowheads point towards membrane surrounded gaps in the blastoderm. (**b**) Confocal scan (maximum intensity projection) of a living stage 2 embryo 30 min after FITC WGA injection. WGA has been heavily endocytosed. (**c**) Lateral view on a living stage 3 embryo stained with FITC-WGA. Smaller germ-disc cells are up, bigger extra-embryonic cells are to the bottom. (**d**) Confocal scan (maximum intensity projection) of a stage 2 embryo stained with an antibody against acetylated tubulin. The arrowhead is pointing towards a cell that is undergoing cell division. (**e**) Living stage 4 embryo stained with FITC-WGA. View on the centre of the germ-disc (the primary thickening). Some of the cone shaped cells of the primary thickening have been coloured in magenta. (**f**) Confocal scan (maximum intensity projection) of a stage 2 embryo stained with DAPI (blue), FITC WGA (green) and phalloidin (red). WGA and phalloidin are co-localized at the cell membrane (arrowheads). In addition, WGA marks the nuclear envelope (arrow). (**g-g''**) An early

stage 3 embryo has been injected with FITC WGA. Dividing cells have been labelled in orange and blue. Scale bar is 25μm in **b**, **c**, **d**, **f** and **g''**.

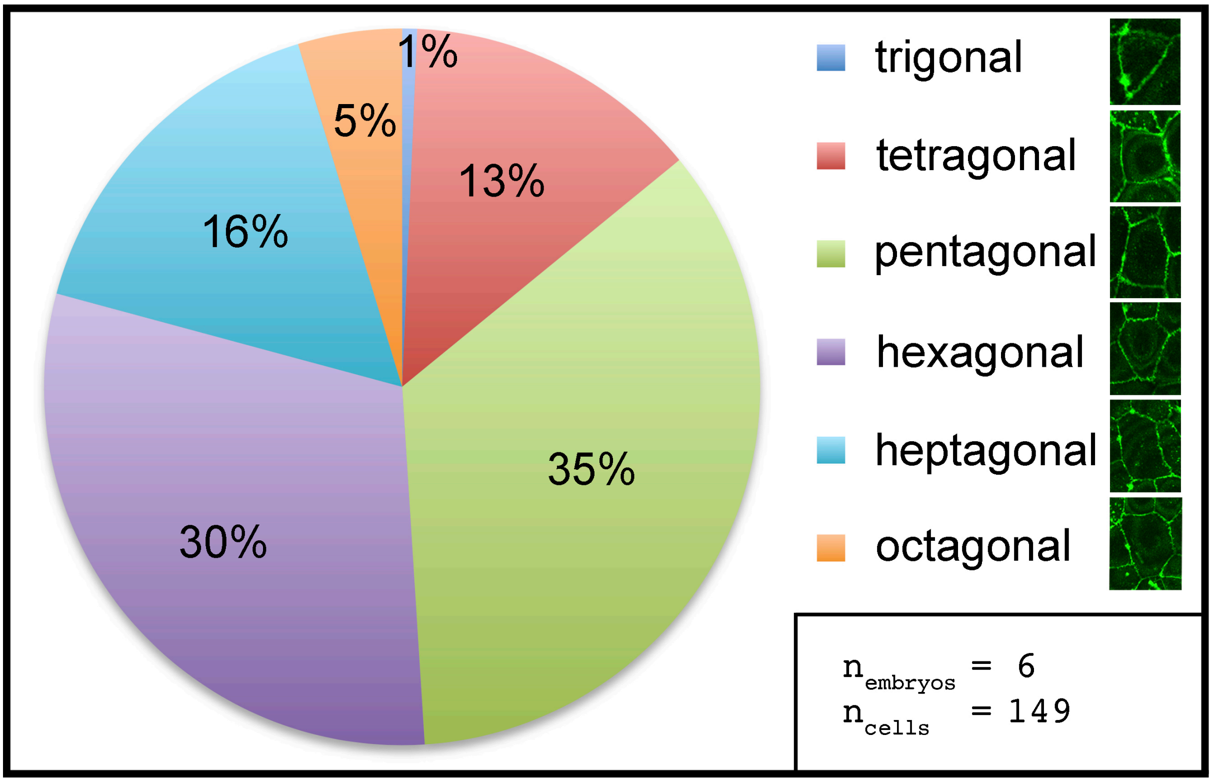

Fig. S2 Cell shapes at late stage 2 . Living embryos have been injected with FITC WGA and maximum intensity projections of confocal scans have been used to analyse the shape of the cells.

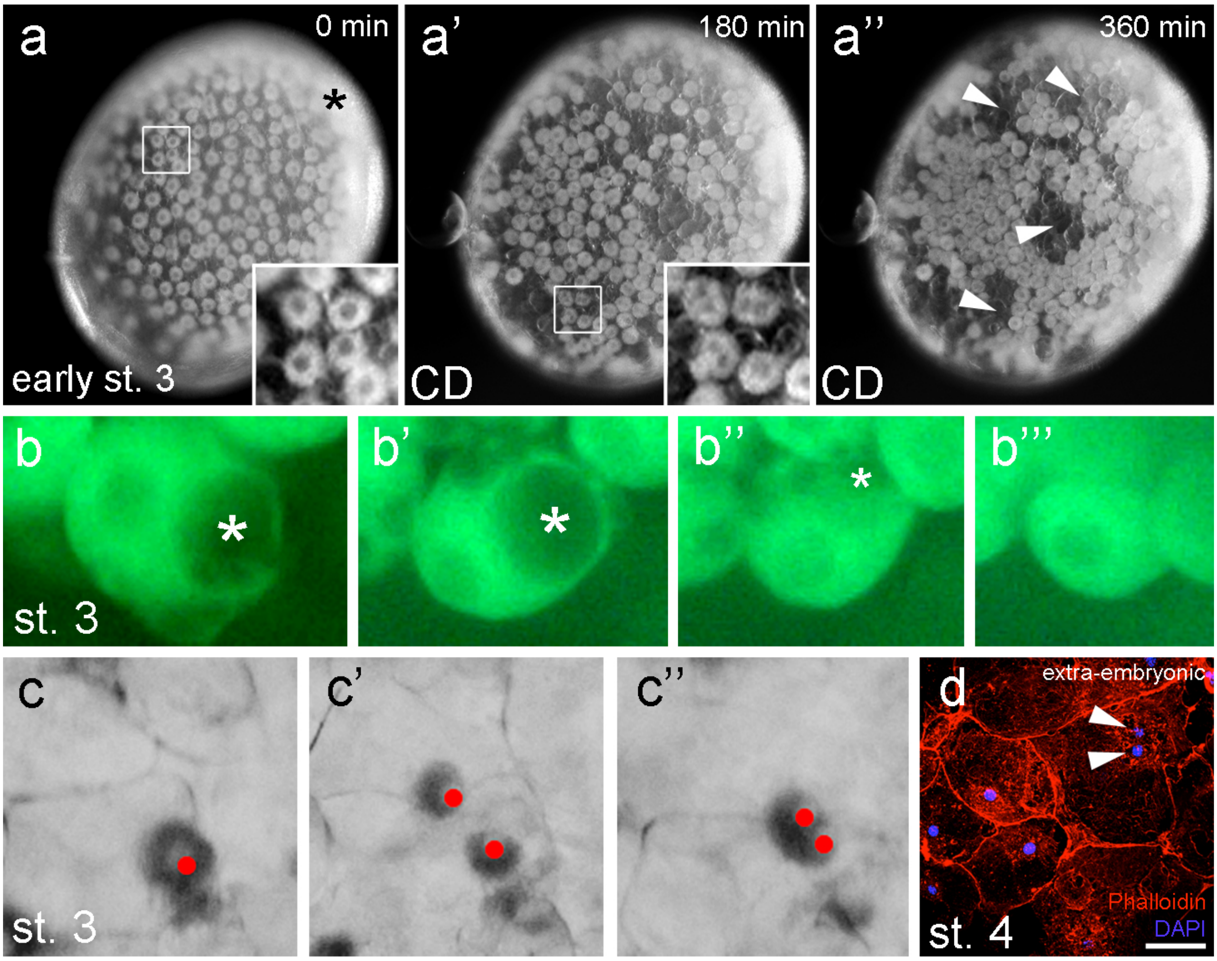

Fig. S3 (**a-a''**) The same embryo before (**a**) and after the injection of cytochalasin D (**a'** and **a''**). The asterisk in **a** indicates the centre of the future germ-disc. After the injection of cytochalasin D (CD) no germ-disc forms and cells cluster. In addition, cytokinesis is disrupted and cells show multiple nuclei (compare insets in **a** and **a'**). Regions of blastoderm disruption are indicated by the arrowheads in **a''**. **a'** and **a''** are stills taken from movie 5. Time corresponds to the time points shown in the movie. (**b-b'''**) Stills from movie 4. During germ disc formation a yolk granule (labelled with an asterisk in **b-b''**) is shifted to a more basal position within or is excluded from the germ-disc cells. (**c-c''**) Tracking of an extra-embryonic nuclei with its attached perinuclear cytoplasm. The nuclei with its attached perinuclear cytoplasm first divide (**c'**) but fuse again (**c''**). This results in an extra-embryonic cell with two nuclei. (**d**) Confocal scan (maximum intensity projection) of extra-embryonic cells stained with phalloidin and DAPI. Two nuclei are present in one cell (arrowheads). Scale bar is 50 μm in **d**.

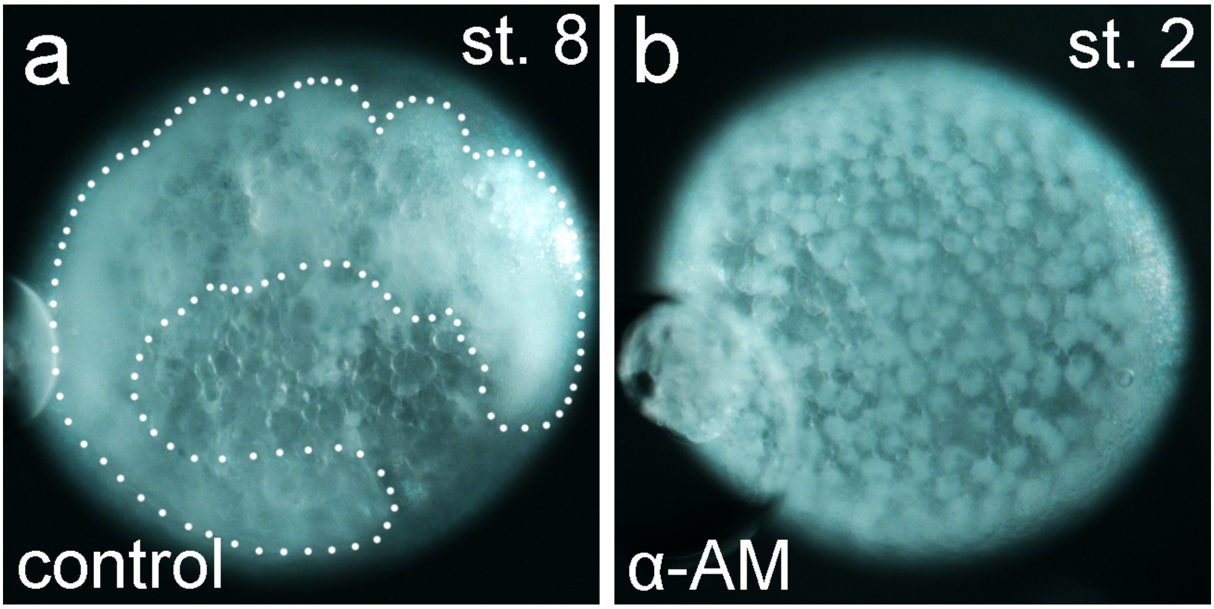

Fig. S4 Two stage 1 embryos of the same cocoon have been injected with water (**a**) or alpha amanitin (**b**). While the control embryo developed normal until germ-band stage, the alpha amanitin injected embryo arrested at stage 2 but was still alive when the control embryo reached stage 8. The development from a stage 2 to a stage 8 embryo takes around two days (see movie 2). The outline of the germ-band has been marked by the dotted line in **a**.

Semi-quantitative-RT-PCR - supplemental Material and Methods

The expression levels of *Pt-hh* and *Pt-PolyUb* has been analysed by running 28, 30, 32 and 35 PCR cycles on cDNA of stage 1, stage 2 and stage 3 embryos. The cDNA was prepared from total RNA that has been extracted separately from stage 1, stage 2 and stage 3 embryos. cDNA concentrations of the different embryonic stages have been adjusted to 1ng cDNA/µl PCR reaction.

To analyse the effect of alpha-amanitin injections on the maternal to zygotic transition, stage 1 embryos of three different spider females (three biological replicates) have been injected with alpha-amanitin or water. The development of the injected embryos has been monitored and the total RNA of around 50 embryos of the water and alpha-amanitin injected embryos has been extracted. For the alpha-amanitin injected embryos only the embryos that got stuck at stage 2 were used for total RNA extractions after the water-injected embryos had reached the beginning of stage 3. cDNA has been prepared from the three water and the three alpha-amanitin RNA samples separately and the absence or presence of *Pt-PolyUb* and *Pt-hh* transcripts has been analysed via PCR.

Primer set used for semi-quantitative-RT-PCR

Shown is the published coding sequence (CDS) for *Pt-hedgehog* (*Pt-hh*) and the partial genome sequence for *Pt-hedgehog*. Primer sequences are in yellow. The different exons are coloured in green and magenta.

1) *Pt-hedgehog*

```
Pt-hh-sqRT-PCR-F: GAG GTG CAA AGA GAA ATT GAA CAC
Pt-hh-sqRT-PCR-R: GAT TTA ACT GAG CAG TGT ATG TGC
```

PCR on cDNA => 261bp
PCR on gDNA => 351bp

>*Pt-hedgehog*
GTTTGAGAGAGATCGGTACACCCATAAATGCCGTCAGTTGAGAATCGAAATGGATGCCATAGACGGGTAG
AACCACAGATTCCAAGGATCGATGTTCCATACTCTGGCAGCCGTACTGTGCCTGGCCGCATTAGTGAATT
CGGCCAGCAGTTGTGGGCCGTTGAGGGGCGGCGGACGACGTAGGCCACCGCGAAAATTAACACCTTTGGT
CTTTAAGCAGCATGTTCCAAACGTGAACGAATTTACGTTAGGTGCAAGTGGTCAGTCTGAAGGAAAATTG
ACCAGAGACCATCCAAAATTTAAAAGCGTGCTTGTTCCAAATTACAATTCGGATATTATATTTCGAGACG
AAGAGGGAACTGGAGCCGATAGACTTATGACACAGAGGTGCAAAGAGAAATTGAACACACTGGCTATTTT
AGTGATGAATCAGTATCCAGGAGTGAAACTTCGCGTCACCGAAGGATTTGACGAAGAGAGCTACCACTCG
ACTCAAAGCTTACACTATGAAGGCAGAGCAGTCGATGTGACCACCAGTGACAGAGACAGAAGCAAGTACG
GAATGCTGGCCAGGCTGGCCGTAGAGGCTGGTTTTGACTTCGTTTATTATGAATCTCGTTCGCACATACA
CTGCTCAGTTAAATCTGAATCTGCTGATGCCGGAAGATCTGGAGGATGTTTCGATGGTGATTCAACAGTT
CGGACAGAAGCTGGGCCAAAGAAAATGTCAGACTTGCAGGTAGGCGAGAGAGTTCAGGTCGCCCGAACAG
ACGGCCAAACAGACTATAGTGAAGTGATTCTGTTTTTGGACAGAAACGAAACACAGCAAAGACTGTACAA
CACCCTAGAGACAGAGAATGGGAGGAGCATCACTCTCACTCCCACGCATCTCATATTCACCGCTTCACCG
CATCAAACGACGCCGCAAGCTACCTTTGCCAAACATGTGGAAATTGGTGATTACATATACGTCGCATCAG
ATCGCAAAGTGACTTTAGAAAAAGTGATCAGCGTTACTAGTTCCGCCAAAAAGGGAGTGTTTGCTCCCTT
GACCAGAGAAGGAAACCTAGTGGTGGATGGTGTAGTAGCTTCCTGCTACGCCATCATAGAAGACCAAGCG
TTGGCACACTTTGCTTTCGCACCGGTCAGACTCATTGACAACGTCTGGGAGGCGACGTTGCATTTGCTAC
GGACTATGCACATCCTCAGGTACAGGGAGAGCAGAACGATACCACCACACAATGGCATACATTGGTACGC
AAACTTTCTCTATTCTATAGCACACAAACTTATACCAGAAGATTGAACTTCCAAAGACGACCGCACTTCA
TAAGAGACCAGAAAAAGAAAAAAGTCAGTCAGAATTTGTGCAATTACAACGCCGAACGCCTGGATTTATG
TATTTCTATACAATTGTCATTTGCAACGGATATAGAATCCTCATTTCCAATTGTAATGCATGGAGACCAA
TGTTAATTGTTTTAGATCATTGTTGCCAAAAATATGGATGGGCACACGATATTTGTAGTGATTGCAACTG
CGTTTTTTTTGACTGATAGCCAACCCGGTAATTTTGTCCACAAATTCGTCTCACATAGCGTAGTCAGAAC
TTTATTTGGTGTGATCTGGCGCTTGTCATGAATATACTTCGAAAATTCTTTTTTTGTAAAGATTATTTAT
TATGTATACAGCGCGAGCTTAGCGAAAAGCTTATTTATTTTATAAGAAAAAGATATATGATGAAAAAACA
AAGAACTTTTCCCTCGAAGTCATGGAGAAGCAGAAGAAATCTTTTGCGTAATTATGTGATTACTGTAATA
TATGTTTGAATATATCAAATATCTATTTTCATGTTGAAATGCACATAACTAAGTTAACTATCTGTATTTG
TAAGAAAAAAAATAATAAAAACTTTTTATCTTTGTCAAAAAAAAAAAAAAA

>Genome-Sequence-*Pt-hedgehog* (Scaffold547)
...
GCTCAACGGATGCACGTCTATTCGCCCAAACGCATGTCCGTCCTTCTGTTATCTATGGCGCTCCACATTTGCTAC
GTCGCAGGGTTTTGGAATTTTATTTTTGGCCAAGGATTTTAACACTAAACATACCAACACTTAATCTATACCTAT
TTCTACCATAGCCGGCCAAATAACCCGACTTTATGTTTCTTGATTGAATGTATGAAATATGGCTGTTAGGAAGGA
AATAAACTAAAAAGAACTTTATTATAATTAAACAAAATTGTATATTGCCAATTTTTATGTATTGCAAACACAAAG
AATAAGGAATTTTATTTCATTGCTCAACGCATTTTTTCCATATACAGTACATTGCGCATTTTCCGCAAACATATT
TCCTCTAATTATTATTATTTCTATAATTCTTATAATTATAAGAATATATAAAAAATATAATTCTTATAATTAATA
TTATTTCTTCTAAGAATTTGTGCTGTCTTGATTGTTTCGACATTTTTGTGGATAAAAACAGTTAAAACAAAGTAA
AGCTACCAAAACATTTAGTTTTTGTCACTTTTTCTGAGAGAAAAATGCCAATCTAAAATTTTAAGTGCTTTCTTC
AAATTTGAATTCACAGTTACTCTGATTGAACTCGCTACGCAACAGCCTTTGAAGAAATGTACAACTCATCGAATG
CAATACGTTGAACACGCATTGCATCGTCATTTCTGATCCGATCACCTTATAAAGTAATAAATTGTTCTCCAGGGT
CAAATGACCGGTTGCGGTAGAAATAGGTAAACGACAAGTATTATTCGAAACAAACAAAATATTAAAAAAATAATA
ATAATAGAATATTTACTGTTATAATTGTTAGAAAAAGTCATGAAGTTAAATGTGTAAAAACGATAAGATGACGAT
CGCATTTTAGATGCGAAAGTTTTTAACGGTCATTTGGCCGATTATGGTAATGTTAAAATTATTTTCGAAGAGTG
GTGATTTTTCAGGCATCACAAGTCTTTTAGCTTGAGTCCAAAATAAAAAAAAAACTATCTTGTTATGAAATAATG
TCTCGATGAGTAATCTCTTCTCTTTTGTGTTTTCAGAGGTGCAAAGAGAAATTGAACACACTGGCTATTTTAGTG
ATGAATCAGTATCCAGGAGTGAAACTTCGCGTCACCGAAGGATTTGACGAAGAGAGCTACCACTCGACTCAAAGC
TTACACTATGAAGGTACGATTATTCATTTTCGAATGTATTGTGATGTCTCCATTTATTTTTGGTATCATTTCATT
GATGTCATTTTTCTTCTCTTTTCAACAGGCAGAGCAGTCGATGTGACCACCAGTGACAGAGACAGAAGCAAGTAC
GGAATGCTGGCCAGGCTGGCCGTAGAGGCTGGTTTTGACTTCGTTTATTATGAATCTCGTTCGCACATACACTGC
TCAGTTAAATCTGGTAAGTGTCCATGACAGTTAACACTGTTCATGACAGTCCATTCACTTTATTGTCCTATCGAT
AAAATGGTGCTAACGATATTTTTAGAATGAGTTTCCAAATATACTGATTTTTATTGGATCATTGGAGTTTTTGAG
GCTTTCTCCAAGATGCACTGACGATACTGACCGTTGAAAACCGACAAAAAACTTAATCGGTTTTTATTTTAAGAG
AAAAATGAAGATATTCCATTCTTCTCTACTTTGTTATAAAGCACACGATTTTGTGTTGAATTAAAATGTTTTGAA
AACGAACTTTTAATTTCGATATAAAATATTTTTCAACGCTGAACGAACAGTATTTTTTGTTGTTACTTTATCCTC
AAATTAAAAAATCACATGAAATGTCAATTTAAAAATTAAGAATTTAATTGGCGCATTTATAATGGTGCAGAACGA
TCAAACTTGATTCAGCAAGAATGTTTTCTAAGTTTCTAAACATTATGAATTCTATTTTATTATTCTTTAAGATTT
TAACTCC
...

2) ***Pt-polyubiquitin-C***

Shown is a part of the CDS for the gene *Pt-polyubiquitin-C* (*Pt-PolyUb*) and the partial genome sequence for *Pt-PolyUb.* Primer sequences are in yellow. The different exons are coloured in blue, grey and turquoise.

```
Pt-PolyUb-sqRT-PCR-F:   CAC ACT TCA TCT TGT CCT GAG G
Pt-PolyUb-sqRT-PCR-R:   CAT TTT CAA TAG TAT CAC TCT GCT C
PCR on cDNA => 110bp
PCR on gDNA => 191bp
```

>*Pt-polyubiquitin-C*

```
GAGGCATGCAAATTTTCGTTAAGACTCTAACAGGAAAAACTATCACTTTAGAGGTAGAGCCCAGTGACAC
AATAGAAAACGTCAAAGCTAAAATTCAAGATAAAGAAGGAATCCCCCCAGATCAGCAAAGGCTTATTTTT
GCCGGAAAACAACTAGAAGATGGTCGAACATTGTCAGACTACAACATACAGAAAGAATCCACACTTCATC
TTGTCCTGAGGCTGCGTGGAGGAATGCAGATCTTCGTTAAAACACTAACAGGCAAGACAATTACTTTAGA
AGTTGAGCAGAGTGATACTATTGAAAATGTCAAAGCAAAAATTCAAGACAAAGAGGGTATCCCTCCCGAT
CAGCAGCGTCTCATTTTTGCCGGCAAGCAACTTGAAGATGGGCGAACCCTTTCAGACTACAACATTCAAA
AGGAATCCACTCTTCATTTGGTACTCCGTCTGAGAGGAGGAATGTAAGGCTGACCAAAATGAATCAGTAA
AGAGTCTTATTCTTCACTTAGTGTTGCGCCTTTATGAAAGGCTGTGATTGCATCAAAAATGAACTTTTTT
TTTTCTTAAATATTTGTGATTAAAGCTAAATTTTGTTTAATTTTGATATTTTAGTATGAGCAGAATAAAG
TTTATTGTTCAAAGCATTAAA
```

>Genome-Sequence-*Pt-polyubiquitin-C* (>Scaffold180)

```
...
GAGGCATGCAAATTTTCGTTAAGACTCTAACAGGAAAAACTATCACTTTAGAGGTAGAGCCCAGTGACACAATAG
AAAACGTCAAAGCTAAAATTCAAGATAAAGAAGGAATCCCCCCAGATCAGCAAAGGCTTATTTTTGCCGGAAAAC
AACTAGAAGATGGTCGAACATTGTCAGACTACAACATACAGAAAGAATCCACACTTCATCTTGTCCTGAGGCTGC
GTGGAGGAATGCAGATCTTCGTTAAAACACTAACAGGTAAGCTTTATGTCCATTTGCTCAAGTGTTTAAATTTGG
CATTTATTTAAATGAAGTTATTTAATTTTGTTTCGTTTTCAGGCAAGACAATTACTTTAGAAGTTGAGCAGAGTG
ATACTATTGAAAATGTCAAAGCAAAAATTCAAGACAAAGAGGGTATCCCTCCCGATCAGCAGCGTCTCATTTTTG
CCGGCAAGCAACTTGAAGATGGGCGAACCCTTTCAGACTACAACATTCAAAAGGAATCCACTCTTCATTTGGTAC
TCCGTCTGAGAGGAGGAATGTAAGGTATATGTAGTTAATGTTTCGTAAATTGTTTTTAAGTTATTTAGTGGTTTT
ATTGTAGAAAAATAAGTTTAAATTTGTATGCGATATTGAGTGTTAAAACTCCGTTCCATGAGGGCTGACAACAAA
AGTACATTTGGCCTTACTTTTTCATTTAGCTATAGGAGCTAAGATTAAGACACTGTAATAACATAATAGAAGAGA
AGGTAATTTTCTTTTGTGAAAGCTGACACATAAATAGCAAAACAAGTCACTAAATTCTTTTAGGGTAGTTCTCGA
AGTATTAAATTTTTGAGATTTCAAACTCTTTTTACCATTTGTCCTGCTGTAAAAGGTCAGTAAAGCCTAATGTCT
CTCCACCTGATTTTTTTAAGAACCGGAAACATATTTGTATTATCTGAACTGCTTGGTCATAATGGTTCTCTAAAT
AGTGGTCAATACGAATAGAATCTCAAAGGAGCTTTCAGAACTCGAAGATGGCACTCCCCAGGATATGAAATATCA
ATATTGTATTGATATATATTGACTCAACATTAAAATTGAATTGATGGCTTAGTATGATGTAGCTTATCTGAATTA
TCAGTTAAACTAATTACATGAAAGAATCTAGTTCAGGGGTATCTGTAAAATTTATTTATTATAAAATTGAACTTC
TAGTATTTAAAGTTTAATGGCAAGCAAGTAAATTATCTAATTTTTTTTAGTAAATATTGTTTCGTAAAAGATTCC
AGAATCTAATGTGAATATGCTGGGATATTTTATATTAAAAGTGCGTTGCATTTTTGAAAGGTGCTTATTTTTGTT
TTTTAAAAGCTAACTGTGTTTTCTTTATGGATTGTTTTTAAAAAATTAAATTATTTTTGAAAATTATTTTTTCTT
TACCTTATCGATTTTTGCTGTGTATTTTGCAAAAGCGTGGTTTCCTAATTGTTCATGCTCTTGGTTGAGATTCTT
GCACTCTCATGTGCAGCTCTGCTATATTTTGCAAGATATTCTCAATTTCAGGTTTCATTTTCTACCCTCTGAAAT
TGAAGCGCAAAATCTATCTTTTTTGGTGGCTAATATAATCAGGAAAAGAGCTCAGTCAGAAACTAGTTGATCACG
ATAATGTAAAGTCTTTGAAAGTTTTGCATTTTGTTAATGTAAATAGTACTTAAGCAACTTAAAGCACTCTAAAAT
ATTTTTATTTTTTGTTCCGATATTTGGCTATGCACCCTGTATGAGTTCGAAATTTTGGTACAAATATCAAAGGTT
ACAAAAGTTTTTTATACTTTAATATTTTATTGGTTCTATGTTAGGTATTATATGTAAATCAGTAGGTATTATTAT
GAAATATCAAATGTTCTATTAAAAAAAAACTAATTACCTTTTTTTTTCTTTCTAGGCTGACCAAAATGAATCAGT
AAAGAGTCTTATTCTTCACTTAGTGTTGCGCCTTTATGAAAGGCTGTGATTGCATCAAAAATGAACTTTTTTTTT
TCTTAAATATTTGTGATTAAAGCTAAATTTGTTTAATTTTGATATTTTAGTATGAGCAGAATAAAGTTTATTGT
TCAAAGCATTAAACCTCTTGGTTTTTATTTTAGCAGTCTTGTTGTAAATGTTAGTTGTAAATGTTTTTGTTATTA
AGGGATGGCTTT
…
```

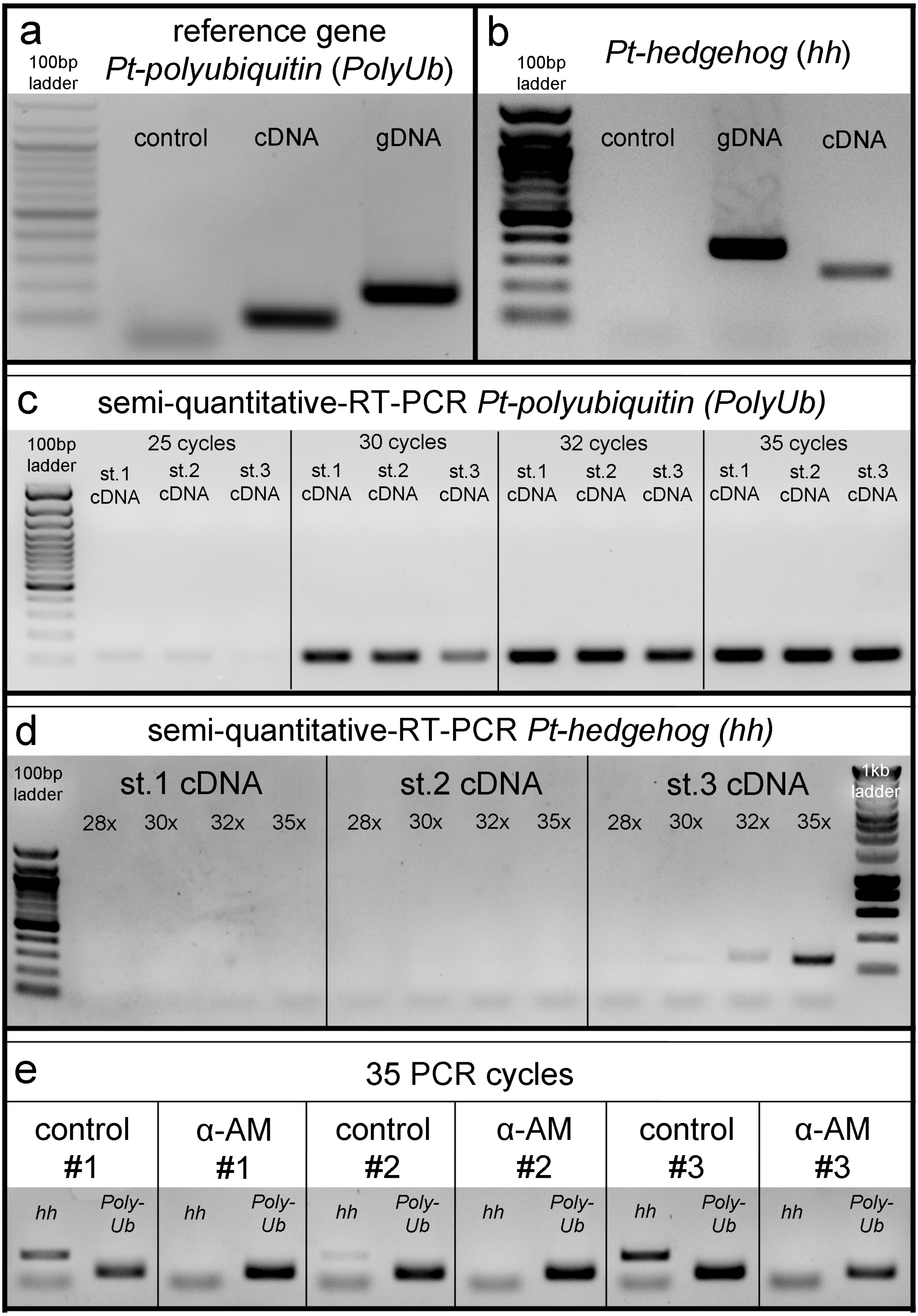

Fig. S5 Gel pictures of PCRs performed with intron spanning primers (see above) for the genes *Pt-PolyUb* and *Pt-hh*. (**a**, **b**) Control PCRs (no template control) and PCRs on genomic (gDNA) and cDNA have been performed (see above for a detailed description of the expected band sizes). The expression of *Pt-PolyUb* in stage 1 (st.1), early stage 2

(st.2) and stage 3 (st.3) embryos has been analysed via semi-quantitative RT PCR (**c**). Compared to stages 1 and 2, *Pt-PolyUb* seems to be expressed at a two fold lower rate at stage 3. *Pt-hh* seems to be not expressed at stage 1 and early stage 2. (**d**) Expression of *Pt-hh* is detectable at stage 3 and a band can be seen best after 35 PCR cycles (35x). Embryos have been injected with water (control #1-3) or alpha-amanitin (α -AM #1-3) and the expression of *Pt-PolyUb* and *Pt-hh* has been analysed by running 35 PCR cycles on the synthesised cDNA samples (three biological replicates). While the reference gene *Pt-PolyUb* is detectable in all samples, *Pt-hh* is only up regulated in the water-injected control embryos (**e**).
